# Time of Day-Dependent Alteration of Hippocampal Rac1 Activation Regulates Contextual Fear Memory in Rats

**DOI:** 10.3389/fnmol.2022.871679

**Published:** 2022-06-16

**Authors:** Lizhu Jiang, Chao Liu, Baizhen Zhao, Chen Ma, Yan Yin, Qixin Zhou, Lin Xu, RongRong Mao

**Affiliations:** ^1^CAS Key Laboratory of Animal Models and Human Disease Mechanisms, KIZ-SU Joint Laboratory of Animal Model and Drug Development, Laboratory of Learning and Memory, Kunming Institute of Zoology, Chinese Academy of Sciences, Kunming, China; ^2^Kunming College of Life Science, University of Chinese Academy of Sciences, Kunming, China; ^3^Department of Clinical Psychology, The Third People’s Hospital of Yunnan Province, Kunming, China; ^4^Department of Pathology and Pathophysiology, School of Basic Medical Science, Kunming Medical University, Kunming, China; ^5^Department of Neuropsychopathy, Clinical Medical School, Dali University, Dali, China; ^6^CAS Centre for Excellence in Brain Science and Intelligent Technology, Shanghai, China

**Keywords:** time of day-dependent, hippocampal Rac1 activity, contextual fear learning, memory retrieval, melatonin

## Abstract

Fear memory in species varies according to the time of the day. Although the underlying molecular mechanisms have been extensively explored, they remain largely unknown. Here, we report that hippocampal Rac1 activity undergoes a time of day-dependent alteration both in nocturnal rats and diurnal tree shrews and that training at the lower hippocampal Rac1 activation period during the night leads to better contextual fear memory in rats. Furthermore, day and night reversion by 24 h darkness/24 h light housing inverses the external clock time of hippocampal Rac1 activation, but the better contextual fear memory still coincides with the lower Rac1 activation in rats during the night. Interestingly, exogenous melatonin treatment promotes hippocampal Rac1 activity and impairs better contextual fear memory acquired at the lower Rac1 activation period during the night, and Rac1-specific inhibitor NSC23766 compromises the effect of melatonin. These results suggest that the time of day-dependent alteration of hippocampal Rac1 activation regulates contextual fear memory in rats by forgetting.

## Introduction

Circadian modulation of learning and memory efficiency exists across species, and time of training is crucial for the efficiency of memory formation and consolidation (Gerstner et al., 2009; Smarr et al., 2014). Previous studies have shown that nocturnal mice acquire T-maze spatial memory, novel object location, and contextual fear conditioning faster in the night-active phase (Hoffmann and Balschun, 1992; Valentinuzzi et al., 2001; Aten et al., 2018). In rats, the acquisition of radial maze, water maze, and novel location recognition is better in the dark phase (Hauber and Bareiss, 2001; Valentinuzzi et al., 2004; Takahashi et al., 2013). However, other studies have shown that mice acquire contextual fear memory better during the inactive day, and diurnal humans display increased fear responses at inactive night (Chaudhury and Colwell, 2002; Li et al., 2015). The molecular mechanisms underlying time-dependent modulation of memory acquisition, consolidation, and retention have been extensively studied, and results show circadian oscillation of MAPK cascade, melatonin, and clock genes such as period and Bmall are involved in time-of-day differences in memory (Sakai et al., 2004; Eckel-Mahan et al., 2008; Rawashdeh and Maronde, 2012). However, little is known about time-of-day differences in memory forgetting.

Rac1 is a member of the Rho family of small GTPases, which plays an important role in synapse formation and plasticity, learning, and memory (Haditsch et al., 2009; Martinez and Tejada-Simon, 2011). Ten years ago, Shuai et al. (2010) first found that Rac1 activity regulated active forgetting in Drosophila. Our previous study also found that hippocampal Rac1 activity regulated the forgetting of contextual fear memory in rats. The activity of Rac1 shifts the balance between memory and forgetting. In this study, we found that better contextual memory acquired at darkness in rats always consistent with lower Rac1-GTP level, therefore, this study elucidated time-dependent Rac1 modulate memory from forgetting viewpoint. Our results show that inhibition of hippocampal Rac1 activity prevents the forgetting of contextual fear memory, while activation of hippocampal Rac1 accelerates the forgetting of contextual fear memory in rats (Jiang et al., 2016b). Recent studies have demonstrated that Rac1 modulates forgetting in various species (Shuai et al., 2010; Jiang et al., 2016b; Liu et al., 2016; Davis and Zhong, 2017). Whether Rac1 activation alters with the time of day and impacts time-of-day differences in memory is unknown. The hippocampus is an important brain area involved in memory processing, including forgetting. Furthermore, the expression and oscillation of various circadian clock genes were observed in the hippocampus (Jilg et al., 2010). It is unclear whether the time of day-dependent mechanisms underlying forgetting also exist in the hippocampus of mammals. Thus, we hypothesized that hippocampal Rac1 activity may undergo a time of day-dependent alteration, contributing to forgetting and time-dependent modulation of contextual fear memory.

In this study, we first explored how hippocampal Rac1 activity is altered depending on the time of the day in both nocturnal rats and diurnal tree shrews during the day. The rats were trained in contextual fear memory at higher or lower points of Rac1 activity to investigate further whether learning and memory efficiency is impacted by forgetting regulated by Rac1 activity. Furthermore, the day and night of rats and tree shrews were inversed by 24 h darkness/24 h light housing to investigate whether hippocampal Rac1 activity is dependent on the external clock time or day and night rhythm. Finally, we explored how exogenous melatonin treatment impacts Rac1 activity and learning and memory efficiency in rats.

## Materials and Methods

### Animals

The adult male Sprague–Dawley rats (250–280 g) were purchased from the Vital River Laboratory Animal Technology Company (Beijing, China). The adult male Chinese tree shrews (*Tupaia belangeri chinensis*) (130–160 g) were obtained from a breeding colony at the Animal House Center of the Kunming Institute of Zoology. All animals were provided with free access to food and water, a 12/12 h light/dark cycle (light, 8:00–20:00; dark, 20:00–8:00), and a thermoregulated environment (T: 25 ± 2^°^C, RH: 55∼70%). The experimental protocols were approved by the Institutional Animal Care and Use Committee of the Kunming Institute of Zoology, Chinese Academy of Sciences (ID: SMKX-20170925-155). All animals were randomly assigned to different experimental and control conditions.

### Experimental Lighting Conditions

Rats and tree shrews were housed in a 12 h light/12 h dark cycle for at least 10 days prior to the start of all experiments. L/D indicates a “normal” light-dark cycle; that is, the time of lights on is 08:00, while the time of lights off is 20:00. Rats were trained during the day (09:00) or night (21:00). D/L indicates a “reverse” light-dark cycle; the light is off until 20:00. The rats were trained during the day (21:00) or night (09:00). For alteration experiments, animals were killed every 4 h during a 12 h-period. In dark lighting conditions, animals were killed under 1 lux. Lux was measured with a light meter (JC, DT-1300).

### Western Blot Analysis

The hippocampal tissues from the rats and tree shrews were harvested at 1, 5, 9, or 13 h after light onset, i.e., 09:00, 13:00, 17:00, or 21:00, and stored in a liquid nitrogen tank for biochemical assay. The tissues were homogenized in a cold lysis buffer (Merck-Millipore, 20–168). The insoluble matter was removed after centrifugation at 13000 rpm (for 10 min at 4°C). For GTP-bound Rac, the supernatant was incubated with GST-tagged PAK-PBD agarose beads (Cytoskeleton, PAK02) for 2 h at 4^°^C. The beads were centrifuged at 5,000 rpm (for 1 min, at 4^°^C) and washed three times with a lysis buffer before being subjected to the GTP-bound Rac and prestained protein ladder (Thermo scientific, 26617) to SDS–PAGE (15%) and transferred onto nitrocellulose membranes (Millipore, ISEQ00010). The membranes containing original blots were blocked for 1 h with TBST (0.9% NaCl, 10 mM Tris, 0.1% Tween-20, pH 7.4) containing 3% BSA (Sigma, B2064) on an orbital shaker at room temperature; Subsequently, the membranes between 17–26 kDa were used for incubating with primary antibody of Rac1 (21 kDa, BD Transduction Laboratories, NO. 610650, 1:2000 dilution) overnight at 4^°^C and with HRP-conjugated goat anti-mouse IgG (Aksomics, KC-MM-035, 1:10000 dilution) for 1 h at room temperature. The total amount of Rac1 in the supernatant was detected using a routine Western blot procedure. Immunoreactivity was detected by using the Gel Imaging System (Tanon, 5200 Multi) with the aid of the Femto-sig ECL western blotting substrate (Tanon, 180–506). The gray value of the detected band in the WB was quantified using ImageJ software (NIH, United States).

### Immunohistochemical Staining

The rats and tree shrews were anesthetized at 09:00 or 21:00 with phenobarbital sodium and perfused with ice-cold 4% paraformaldehyde in 0.01 M phosphate-buffered saline (PBS). The brains were post-fixed for 4h at 4^°^C and dehydrated in 30% sucrose in 0.01 M PBS. Then, the brains were sectioned (40 μm thick coronal sections) using a cryostat and stored in PBS. Following that, Rac1-GTP immunostaining was performed; free-floating sections were placed in a 0.01 M PBS solution containing 5% BSA and 0.3% Triton X-100 for 1 h, followed by incubation with primary antibody mouse anti-active Rac1, i.e., Rac1-GTP (NewEast, 26903, 1:800 dilution) overnight at 4°C, then washed slices three times in PBS, followed by 2 h of incubation with secondary antibody (Donkey anti-mouse Alexa-488, Invitrogen, A21202, 1:1000 dilution) at room temperature. The slices were subsequently washed three times, followed by mounting and cover-slipping on microscope slides. Images were captured using a confocal microscope (Olympus, FV3000) with a 20×objective at the same settings for all conditions.

### Immunohistochemistry Data Analysis

Quantification of immunohistochemical staining of Rac1-GTP was performed in the pyramidal neurons of CA1 by utilizing the imaging analysis function of ImageJ software (Jensen, 2013). The integrated intensity quantification of Rac1-GTP was performed by setting the threshold manually from 30 to 255. Two coronal brain sections per animal were quantified at two different bregma levels (−3.24 and −3.36 mm), and quantitative analysis was obtained at 20× magnification.

### Administration of Drugs

Melatonin (Sigma, M5250-1 G) was dissolved in pure ethanol to the stock solution (100 μg/μl) and diluted in normal saline to the desired concentration (2 μg/μl), and NSC 23766 (TOCRIS, 2161–10 mg) was dissolved in saline to the working concentration (0.5 μg/μl) immediately before administration. Melatonin was administrated intragastrically to naïve rats before contextual fear conditioning learning (10 mg/kg). NSC 23766 was administrated intraperitoneally (2.5 mg/kg) 6 h before contextual fear conditioning learning. All control groups were treated with the vehicle solution.

### Contextual Fear Conditioning

The experiments on rats were performed in the NIR Video Fear Conditioning System for Rat (MED Associates Inc., MED-VFC-SCT-R); the chambers (30 cm × 24 cm × 21 cm) were enclosed in a ventilated and sound-attenuated box (63 cm × 43 cm × 63 cm). The animals were placed into the chamber for 2 mins and before receiving three footshocks at an intensity of 0.5 mA for 2 s. The inter-trial intervals (ITIs) of the three footshocks were 122 s, 2 mins after the last footshock; the animals were placed back into their home cages. When the experiment required training animals during the night or under D/L conditions, all training and subsequent testing took place under dim red light conditions (< 1 lux) (Eckel-Mahan et al., 2008), regardless of the normal time.

### Statistical Analysis

All data were reported as the mean ± SEM. All analyses were performed using GraphPad Prism 8.3. Comparisons were made with an unpaired *t*-test to compare Gaussian distributions, while Mann–Whitney tests were used for no Gaussian distributions. We used one-way ANOVA and two-way ANOVA with repeated measures (RM), followed by Bonferroni’s post-tests for multiple comparisons where appropriate. Results were deemed statistically significant when the *p*-value < 0.05.

## Results

### Hippocampal Rac1 Activity Fluctuates Oppositely in Nocturnal Rats and Diurnal Tree Shrews During the Daytime

**FIGURE 1:**
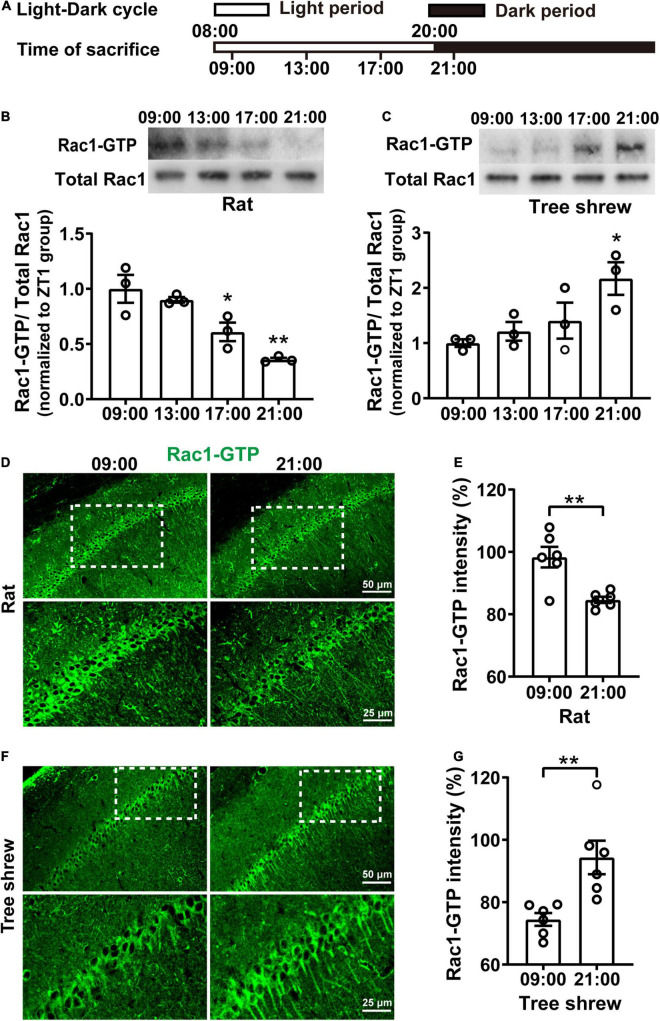
Rac1 activity in the hippocampus shows fluctuates oppositely in nocturnal rats and diurnal tree shrews during the daytime. **(A)** Diagram for experimental procedures. The light was turned on at 08:00 and turned off at 20:00. Rats and tree shrews were sacrificed at 09:00, 13:00, 17:00, or 21:00. **(B)** Rac1 activity in the hippocampus shows gradually decreased from 09:00 to 21:00 in rats. Upper panel: representative WB demonstrated that Rac1 activity sharply decreased at the 17:00 and 21:00 groups compared with the 09:00 group. Lower panel: statistical bar graph showed an obvious inhibition of Rac1-GTP in the 17:00 group and 21:00 group compared with the 09:00 group. **(C)** Rac1 activity in the hippocampus shows gradually increased from 09:00 to 21:00 in tree shrews. Upper panel: representative WB demonstrated that Rac1 activity remarkably increased in the 21:00 group compared with the 09:00 group. Lower panel: statistical bar graph showed an obvious increase of Rac1-GTP in the 21:00 group compared with the 09:00 group. **(D)** Representative images of IHC for Rac1-GTP in rats. **(E)** The statistical bar graph showed that the integrated density of Rac1-GTP in the CA1 area was significantly decreased in the 21:00 group compared with the 09:00 group. **(F)** Representative images of IHC for Rac1-GTP in tree shrews. **(G)** The statistical bar graph showed that the integrated density of Rac1-GTP in the CA1 area was significantly increased in the 21:00 group compared with the 09:00 group. Data are presented as mean ± SEM, *p < 0.05, **p < 0.01.

To determine whether hippocampal Rac1 activity fluctuated during the daytime, both nocturnal rats and diurnal tree shrews maintained a 12-h light/12-h dark (L/D) cycle for 10 days. Then, their hippocampal tissue was collected every 4 h, starting from 1 h after lights were on (08:00) and ending at 1 h after lights were off (21:00) (Figure 1A). In particular, rats and tree shrews were killed at 21:00 under red light to avoid light-induced effects. Rac1 activity showed a pronounced oscillation in the opposite direction in both rats and tree shrews over the 12-h period both, as determined by western blot analysis. To determine whether activated Rac1 fluctuated relative to total Rac1 protein, we quantified Rac1-GTP levels relative to total Rac1 protein. Total Rac1 was kept invariant in all groups. Rac1-GTP sharply decreased at 17:00 and 21:00 groups compared with 09:00 group in rats [Figure 1B, *N* = 3 for per group; *F*_(3, 8)_ = 14.07, *p* = 0.0015; 09:00 vs. 13:00, *p* > 0.9999; 09:00 vs. 17:00, *p = 0.0220; 09:00 vs. 21:00; ^**^p = 0.0011; One-way ANOVA followed by Bonferroni’s post-tests]. In tree shrews, Rac1-GTP increased remarkably at 21:00 group compared with 09:00 group [Figure 1C, *N* = 3 for per group; *F*_(3, 8)_ = 4.583, *p* = 0.0378; 09:00 vs. 13:00, *p* > 0.9999; 09:00 vs. 17:00, *p* = 0.7844; 09:00 vs. 21:00; *p = 0.0251; One-way ANOVA followed by Bonferroni’s post-tests]. These results indicated that hippocampal Rac1 activity alteration was dependent on the time of day. Interestingly, Rac1-GTP expression showed a decreased direction from light on to light off in nocturnal rats but showed an increased direction in diurnal tree shrews. Because there was a significant difference in Rac1-GTP in the 09:00 group and the 21:00 group, both in rats and tree shrews, the two time points were used for subsequent experiments.

Immunofluorescence staining of hippocampal slices was carried out to investigate further whether Rac1-GTP expression in the CA1 area fluctuated during the daytime in rats and tree shrews at 09:00 and 21:00 time points; we observed Rac1-GTP staining in the pyramidal layer of areas of CA1. In rats, Rac1-GTP expression decreased significantly in the 21:00 group compared to that in the 09:00 group (Figures 1D,E, for Figure 1E, *n* = 6 (*N* = 3) for per group; *t* = 3.966, df = 10, ^**^p = 0.0027; unpaired *t*-test). In tree shrews, Rac1-GTP expression increased significantly in the 21:00 group compared to that in the 09:00 group (Figures 1F,G, for Figure 1G, *n* = 6 (*N* = 3) for per group; *t* = 3.455, df = 10, ^**^p = 0.0062, unpaired *t*-test). These results were consistent with those in the western blot.

These results show that Rac1 activity altered depending on the time of day both in rats and tree shrews. Previous evidence has demonstrated that Rac1 activity regulates learning and memory by facilitating forgetting. Whether the forgetting also altered during the daytime with Rac1 activation remains to be studied. Higher Rac1 activation leads to bad memory, while the lower Rac1 activation leads to good memory.

### Training at the Lower Hippocampal Rac1 Activation Period During the Night Leads to Better Contextual Fear Memory in Rats

**FIGURE 2:**
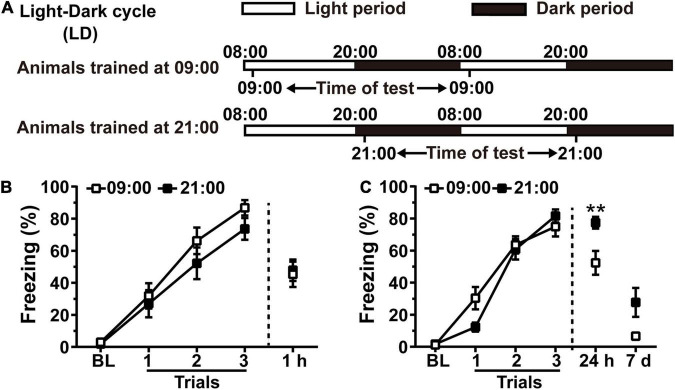
Training at the lower hippocampal Rac1 activation period during the night leads to better contextual fear memory in rats. **(A)** Diagrams for experimental procedures. **(B)** The contextual fear memory was not different between the 09:00 group and 21:00 group when tested at 1 h after learning. **(C)** The contextual fear memory was better in the 21:00 group at 24 h and 7 days after learning. Data are presented as mean ± SEM. **p < 0.01.

Because inhibition of hippocampal Rac1 activity prevents forgetting of contextual fear memory in rats (Jiang et al., 2016b), we speculated that there is a correlation between alterations of hippocampal Rac1 activity and forgetting of contextual fear memory. Rats were housed in an L/D cycle for 10 days. On the 11th day, animals were divided into the 09:00 group and the 21:00 group, which received contextual fear conditioning at the higher or lower hippocampal Rac1 activation period, and contextual fear memory was tested at 1 h or 24 h after training. Animals tested at 24 h received a retest at 7 days after training (Figure 2A). Contextual fear memory in the 21:00 group was similar to that in the 09:00 group at 1 h after training [Figure 2B, *N* = 11 for per group; for learning curve, group, *F*_(1, 60)_ = 1.422, *p* = 0.2471; trial, *F*_(3, 60)_ = 79.17, *p* < 0.0001; trial × group, *F*_(3, 60)_ = 0.6803, *p* = 0.5676; Two-way RM ANOVA followed by Bonferroni’s post-tests; for 1 h, *t* = 0.2442, df = 20, *p* = 0.8096, Unpaired *t*-test]. However, 24 h after training, the freezing time of the 21:00 group was significantly higher than the 09:00 group. The 7 days retest results showed that freezing time in the 21:00 group still kept better compared to that in the 09:00 group [Figure 2C, *N* = 13 for per group; for learning curve, group, *F*_(1, 72)_ = 0.4818, *p* = 0.4943; trial, *F*_(3, 72)_ = 178.5, *p* < 0.0001; trial × group, *F*_(3, 72)_ = 3.917, *p* = 0.012; Two-way RM ANOVA followed by Bonferroni’s post-tests; for 24 h: *t* = 2.990, df = 24, *p* = 0.0064, Unpaired *t*-test; for 7 days: *p* = 0.2642, Mann–Whitney test]. All groups showed no difference in the acquisition of contextual fear conditioning demonstrated by the learning curve and 1 h memory test. The 21:00 group showed higher memory performance at both 24 h and 7 days retest, which is consistent with our speculation that lower Rac1 activation leads to better memory. These results have demonstrated that time of day-dependent alteration of hippocampal Rac1 activation impacts contextual fear memory in rats.

### The Better Contextual Fear Memory Still Coincides With the Lower Rac1 Activation in Rats During the Night After Day and Night Reversion

**FIGURE 3:**
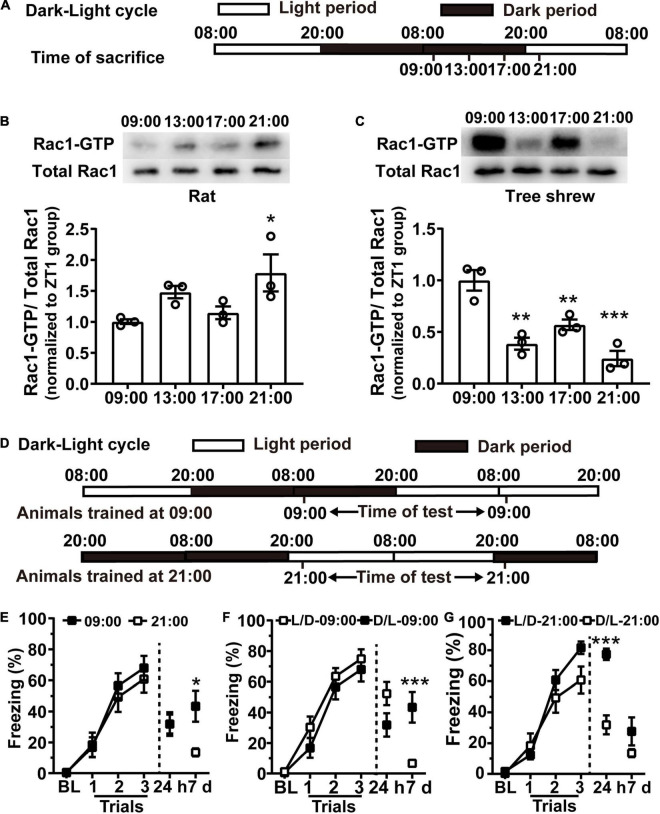
The better contextual fear memory still coincides with the lower Rac1 activation in rats during the night after day and night reversion. **(A,D)** Diagrams for experimental procedures. **(B)** Acute light irradiation shifts the hippocampal Rac1 activation cycle in rats. WB demonstrated that Rac1 activity remarkably increased in the 21:00 group compared with the 09:00 group. **(C)** Acute alteration of light conditions shifts the hippocampal Rac1 activation cycle in tree shrews. WB demonstrated that Rac1 was sharply activated at 09:00 compared with the 21:00 group. **(E)** The freezing in the 09:00 group was similar to the 21:00 group in 24 h test and significantly higher at 7 days after training under the D/L cycle. L/D represents the light-dark cycle, D/L represents the dark-light cycle. **(F)** The 09:00 group under D/L cycle presented significantly higher freezing than the 09:00 group under L/D cycle at 7days after learning. **(G)** The 21:00 group under L/D cycle presented significantly higher freezing than the 21:00 group under D/L cycle at 24 h and 7 days after learning. Data are presented as mean ± SEM. *p < 0.05, **p < 0.01, ***p < 0.001.

To confirm whether training at the lower hippocampal Rac1 activation period is sufficient for better contextual fear memory, we first attempted to modulate Rac1 activity in the hippocampus by changing external light illumination. As light is a key factor affecting the endogenous system in mammals, we altered the light illumination condition of the animals by exposing them to 24 h of darkness, followed by 24 h of light. Animals were housed in the L/D cycle for 10 days, and on the 11th day, the light was turned off from 20:00 to 20:00 the next day, followed by 24 h of light exposure (Figure 3A). Hippocampal tissues were harvested at 09:00, 13:00, 17:00, and 21:00 on the 12th day and analyzed Rac1-GTP by western blotting. Both rats and tree shrews were killed at 09:00, 13:00, and 17:00 under red light to avoid light-induced effects. Western blotting results showed the Rac1 activation period reversed under this condition compared to the normal L/D condition. In rats, the higher Rac1 activity occurred at 21:00 during the light time, and the lower Rac1 activity occurred at 09:00 during the dark time. One-way ANOVA analysis showed that hippocampal Rac1 activity significantly increased in the 21:00 group compared to the 09:00 group [Figure 3B, *N* = 3 for per group; *F*_(3, 8)_ = 4.451, *p* = 0.0405; 09:00 vs. 13:00, *p* = 0.2344; 09:00 vs. 17:00, *p* > 0.9999; 09:00 vs. 21:00; *p = 0.0310; one-way ANOVA followed by Bonferroni’s post-tests]. In tree shrews, the higher hippocampal Rac1 activity occurred at 09:00 and the lower presented at 21:00. One-way ANOVA analysis shows there was an obvious difference between the groups; hippocampal Rac1 activity in the 13:00 group, 17:00 group, and 21:00 group decreased sharply compared to the 09:00 group [Figure 3C, *N* = 3 for per group; *F*_(3,8)_ = 20.02, *p* = 0.0004; 09:00 vs. 13:00, ^**^p = 0.0011; 09:00 vs. 17:00, ^**^p = 0.0097; 09:00 vs. 21:00; ^***^p = 0.0003; One-way ANOVA followed by Bonferroni’s post-tests]. In addition, we noted that there are apparent bimodal Rac1 activation profiles for both rats and tree shrews under the D/L cycle (Figures 3B,C). The second peaks at 13:00 for rats and 17:00 for tree shrews hinted besides light, the Rac1 activation cycle is regulated by endogenous circadian rhythms. These results have demonstrated that the time of day-dependent alteration of hippocampal Rac1 activation can be regulated by the environmental light-dark cycle. Interestingly, hippocampal Rac1 activation decreased in a dark environment but increased in a light environment in nocturnal rats. Unlike nocturnal rats, in diurnal tree shrews, hippocampal Rac1 activation decreased in light.

Next, we detected whether the alteration of hippocampal Rac1 activity regulated by the light-dark cycle could impact contextual fear conditioning in rats. The rats were entrained in an L/D cycle for 10 days before undergoing 24 h of darkness, followed by 24 h of light, as shown in the western blotting experiments above. On the 11th day, the rats received contextual fear training at 09:00 or 21:00; the contextual fear memory was examined 24 h after training and retested 7 days later. We found that the learning curve and contextual fear memory in the 09:00 group were similar to those in the 21:00 group when retrieved 24 h after training, but the 09:00 group performance was significantly higher than in the 21:00 group after the 7-days retest [Figure 3E, *N* = 11 for per group; for learning curve, group, *F*_(1, 60)_ = 0.1730, *p* = 0.6819; trial, *F*_(3, 60)_ = 68.85, *p* < 0.0001; trial × group, *F*_(3, 60)_ = 0.4104, *p* = 0.7461; Two-way RM ANOVA followed by Bonferroni’s post-tests; for 24 h: *t* = 0.0072, df = 20, *p* = 0.9943; for 7 days: *t* = 2.885, df = 20, ^**^p = 0.0092, unpaired *t*-tes].

We also compare fear memory acquired at same time point but under light time (L/D) or dark time (D/L). The learning curve and 24 h memory in D/L-09:00 group were similar to those in the L/D-09:00 group, but in the 7 days memory retrieval, the freezing of D/L-09:00 group was significantly higher than in the L/D-09:00 group [Figure 3F, L/D-09:00 group, *N* = 13; D/L-09:00 group, *N* = 11; for learning curve, group, *F*_(1, 66)_ = 1.263, *p* = 0.2732; trial, *F*_(3, 66)_ = 104, *p* < 0.0001; trial × group, *F*_(3, 66)_ = 0.6731, *p* = 0.5716; two-way RM ANOVA followed by Bonferroni’s post-tests; for 24 h: *t* = 1.903, df = 22, *p* = 0.0701; unpaired *t*-test; for 7 days: *p* = 0.0024; Mann–Whitney test]. Similarly, the L/D-21:00 group was higher than D/L-21:00 group in the 24 h memory retrieval [Figure 3G, L/D-21:00 group, *N* = 13; D/L-21:00 group, *N* = 11; for learning curve, group, *F*_(1, 66)_ = 1.154, *p* = 0.2943; trial, *F*_(3, 66)_ = 119.4, *p* < 0.0001; trial × group, *F*_(3, 66)_ = 3.802, *p* = 0.0141; two-way RM ANOVA, followed by Bonferroni’s post-tests; for 24 h: *t* = 6.565, df = 22, *p* < 0.0001; unpaired *t*-test; for 7 days: *p* = 0.9547; Mann–Whitney test].

These results demonstrated that the lower hippocampal Rac1 activation appears during the dark time in nocturnal rats, resulting in enhanced learning and memory during their active period to habituate environment.

### Melatonin Treatment Promotes Hippocampal Rac1 Activity and Impairs the Better Contextual Fear Memory Acquired at the Lower Hippocampal Rac1 Activation Cycle

The above results showed that light plays a key role in the time of day-dependent alteration of hippocampal Rac1 activity period. Melatonin is a hormone released by the pineal gland at night; its synthesis is affected by light (Lewy et al., 1980) and plays an important role in memory processing (Rawashdeh and Maronde, 2012). Thus, we hypothesized that melatonin might be a pivotal molecule in regulating hippocampal Rac1 activity.

**FIGURE 4:**
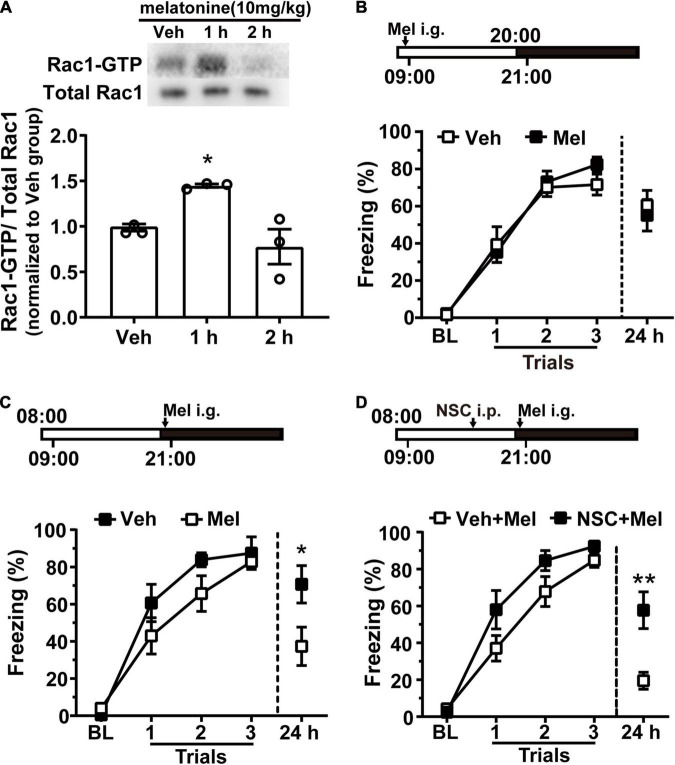
Melatonin treatment promotes hippocampal Rac1 activity and impairs the better contextual fear memory acquired at the night. **(A)** Melatonin activated hippocampal Rac1. Upper panel: representative WB demonstrated that melatonin obviously activated Rac1 at 1 h after administration. Lower panel: the statistical bar graph showed an obvious activation of Rac1-GTP in the 1 h group compared with the vehicle group. **(B)** There was no difference between the vehicle and melatonin group in learning curve and 24 h memory test when training at 09:00. **(C)** The learning curve of the vehicle group and melatonin group was similar when training at 21:00 but melatonin treatment impaired contextual fear memory tested in 24 h after training. **(D)** Inhibition of Rac1 activity compensated for the effect of melatonin on contextual fear memory when training at the night. Contextual fear memory was significantly increased in the NSC + Mel group compared to the Vel + Mel group at 24 h after learning. Data are presented as mean ± SEM. *p < 0.05, **p < 0.01.

Melatonin (10 mg/kg) was given to rats by intragastric administration at 21:00. One or two hours later, the hippocampal tissue was harvested, and Rac1 activity was detected using western blotting. Melatonin significantly promoted hippocampal Rac1 activity 1 h after administration [Figure 4A, *N* = 3 for per group; *F*_(2, 6)_ = 9.243, *p* = 0.0147; Veh *vs.* 1 h, *p = 0.0487; Veh vs. 2 h, *p* = 0.5632; one-way ANOVA, followed by Bonferroni’s post-tests]. Next, we detected whether melatonin could regulate contextual fear memory by enhancing hippocampal Rac1 activity. Rats were entrained to an L/D cycle for 10 days. First, rats received contextual fear conditioning training at 09:00 on the 11th day.10 mg/kg melatonin was given by intragastric administration before training at 9:00 when melatonin level was low and Rac1 activity was high. There was no difference between the vehicle and melatonin group in the learning curve and the 24-h memory test [Figure 4B, Veh, *N* = 12; Mel, *N* = 13; for learning curve, group, *F*_(1, 69)_ = 0.2557, *p* = 0.6179; trial, *F*_(3, 69)_ = 117.8, *p* < 0.0001; trial × group, *F*_(3, 60)_ = 0.9517, *p* = 0.4206; two-way RM ANOVA, followed by Bonferroni’s post-tests; for 24 h, *t* = 0.4731, df = 23, *p* = 0.6406; unpaired *t*-test]. It is possible that melatonin cannot further promote Rac1 activity when Rac1 activity is high. Thus, we administrated melatonin at 21:00 when Rac1 activity began to drop. Melatonin was given by intragastric before training, and contextual fear memory was examined 24 h later. The results showed that contextual fear memory in the melatonin group was significantly impaired compared to the vehicle group [Figure 4C, *N* = 9 for per group; for learning curve, group, *F*_(1, 48)_ = 2.36, *p* = 0.144; trial, *F*_(3, 48)_ = 64.2, *p* < 0.0001; trial × group, *F*_(3, 48)_ = 1.317, *p* = 0.2798; two-way RM ANOVA, followed by Bonferroni’s post-tests; for 24 h, *p = 0.0315; Mann–Whitney test]. These results demonstrated that exogenous melatonin impaired contextual fear memory by enhancing hippocampal Rac1 activation.

In a previous study, we found that hippocampal Rac1 activity sharply decreased 1 h after contextual learning, and Rac1 specific inhibitor NSC23766 significantly inhibited hippocampal Rac1 activity 6 h after administration (Jiang et al., 2016b). Therefore, rats were intraperitoneally administrated Rac1-specific inhibitor NSC23766 (2.5 mg/kg) or vehicle 5 h before training. Further, immediately before training, melatonin (10 mg/kg) was given by intragastrical administration. Results show the learning curves were similar between the NSC + Mel group and the Veh + Mel group, but the effect of melatonin on 24 h memory test was blocked in the NSC + Mel group, indicating by significantly increased freezing relative to that in the Veh + Mel group at 24 h after training. [Figure 4D, *N* = 10 for per group; for learning curve, group, *F*_(1, 54)_ = 3.789, *p* = 0.0647; trial, *F*_(3, 54)_ = 111, *p* < 0.0001; trial × group, *F*_(3, 60)_ = 1.988, *p* = 0.1266; two-way RM ANOVA, followed by Bonferroni’ s post-tests; for 24 h, *t* = 3.488, df = 18, ^**^p = 0.0026, unpaired *t*-test]. These data demonstrated that the Rac1-specific inhibitor NSC23766 compensated for the effect of melatonin, which indicates that exogenous melatonin modulates fear memory through regulating hippocampal Rac1 activity in rats.

## Discussion

The circadian regulation of learning and memory exists across species, from aplysia to mice, rats, and humans, appearing as significant alterations in memory efficiency with the time of the day (Gerstner and Yin, 2010; Albrecht and Stork, 2017). Studies show circadian oscillations of memory efficiency are related to several molecules such as PER, MAPK, CREB, and hormones such as melatonin (Eckel-Mahan et al., 2008). These studies mainly focus on memory acquisition, consolidation, or retention. However, little is known about the role of forgetting in the time-of-day difference in memory. Here, we first revealed that forgetting-related molecular hippocampal Rac1 activation alters depending on the time of day. Rac1-GTP expression showed a decreased direction from light on to light off in nocturnal rats but showed an increased direction in diurnal tree shrews. Furthermore, in nocturnal rats, we have demonstrated that training at the lower hippocampal Rac1 activation during the night leads to better contextual fear memory. Interestingly, we found that exogenous melatonin can increase hippocampal Rac1 activity and impair contextual fear memory acquired at the lower Rac1 activation during the night, while the Rac1-specific inhibitor NSC23766 compensated for the effect of melatonin. These findings suggest that time of day-dependent alteration of hippocampal Rac1 activation impacts contextual fear memory in rats.

Studies show that animals always learn better during their active phase (Decker et al., 2007; Rawashdeh et al., 2007; Harrison et al., 2017). Our results showed that rats acquired better contextual fear memory during the night but got worse during the day. Consistent with our results in contextual fear memory, previous results showed that nocturnal mice acquire T-maze spatial memory, novel object location, and contextual fear conditioning faster in the night-active phase (Hoffmann and Balschun, 1992; Valentinuzzi et al., 2001; Aten et al., 2018). In rats, the acquisition of radial maze, water maze, and novel location recognition is better in the dark phase (Hauber and Bareiss, 2001; Valentinuzzi et al., 2004; Takahashi et al., 2013). However, other studies have shown that mice acquire and recall contextual tone-cued fear memory better during the inactive day, while diurnal humans display increased fear responses at inactive night (Chaudhury and Colwell, 2002; Li et al., 2015). These differences in the timing of learning efficiency are likely stimulus-specific. Chaudhury and Colwell (2002) use different training protocols in mice, including tone-cued fear conditioning training and testing of both tone-cued and contextual fear memory, in which the hippocampus and amygdala are involved. In our experiment, we use contextual fear memory, which is more related to the hippocampus. Different time-of-day effects on fear memory may also be affected by the sleep and behavioral state during training. These findings provide the possibility that different cognition behaviors may achieve the best efficiency at different times of the day (Smarr et al., 2014).

Rac1 is a member of the Rho family of small GTPases, which plays an important role in synaptic plasticity and memory (Martinez and Tejada-Simon, 2011; Dietz et al., 2012). Previous studies have shown that Rac1 is abundantly expressed in the hippocampus (Olenik et al., 1997). Rac1-dependent forgetting plays a vital role in memory processes, including acquisition, consolidation, and extinction across species (Haditsch et al., 2009; Davis and Zhong, 2017). Our previous study reported that lower hippocampal Rac1 activity makes spaced learning more efficient than massed learning in contextual fear learning (Jiang et al., 2016b), and increased hippocampal Rac1 activity impairs the spatial learning in Morris water maze in aged APP/PS mice (Wu et al., 2019). Astrocytic Rac1 activity in the basolateral amygdala (BLA) is required to acquire conditioned fear memory (Liao et al., 2017). Inhibition of hippocampal Rac1 activity can impair the extinction of contextual fear memory, object recognition memory and social memory (Jiang et al., 2016a; Liu et al., 2016, 2018). Several pieces of evidence also suggest that Rac1 is involved in memory consolidation. Rac1 activation in BLA impacts the consolidation of conditioned fear in mice (Gao et al., 2015; Das et al., 2017). The present study first reported that hippocampal Rac1 activity altered depending on the time of day in both diurnal tree shrews and nocturnal rats. The higher Rac1 activation appeared in nocturnal rats or diurnal tree shrews during their inactive period, while the lower Rac1 activation occurred during their active period. Rats acquired better contextual fear memory during the night at the lower hippocampal Rac1 activation; conversely, they got worse contextual fear memory during the day at higher Rac1 activation. How does the time-of-day dependent alteration of hippocampal Rac1 activation impact the processes of contextual fear memory? Our results showed that the acquisition of contextual fear is not affected, as indicated by the intact learning curves. The impaired 24-h test performance may be affected by the impaired consolidation or retrieval, which our data cannot distinguish. In future work, we will provide more evidence to implicit this question by interfering with the Rac1 activation at different time points. These results suggested that hippocampal Rac1 activation is coincidental in rats or tree shrews’ activity rhythms. Our result has provided a Rac1-dependent forgetting mechanism underlying better memory recall in nocturnal rats during the active phase. In addition, we will provide evidence for tree shrews’ memory in our future study to confirm this finding.

Disruption of circadian rhythm impairs learning and memory; the phenomenon has been recognized for decades. Photoperiod shifting is a method for modeling circadian rhythm disruption (Craig and McDonald, 2008; Zelinski et al., 2014). Loh et al. (2010) found that acute changes in the light-dark cycle disrupt contextual fear memory in mice, Here, day and night inversion by 24 h darkness/24 h light housing inverses the external clock time of hippocampal Rac1 activation in both rats and tree shrews, and we observed there are apparent bimodal Rac1 activation profiles for both rats and tree shrews under the acute altered light-dark cycle, but the better contextual fear memory still coincides with the lower Rac1 activation during the night time in rats. These results indicate that the alteration of the hippocampal Rac1 activity cycle is regulated by the activity-inactivity rhythm of rats and tree shrews but still regulated by the environmental cue, such as light. The lower hippocampal Rac1 activation can improve the learning and memory efficiency of rats during their active period when they deal with a more complex environment.

Melatonin is a hormone released by the pineal gland at night, and its synthesis is affected by light (Lewy et al., 1980) and plays an important role in the circadian modulation of memory processing (Rawashdeh and Maronde, 2012; Jilg et al., 2019). The relationship between melatonin and Rac1 activity is still unclear. In the present study, we first found that exogenous melatonin administration can increase hippocampal Rac1 activity. It is well known that light suppresses the secretion of melatonin. The endogenous melatonin gradually reaches its peak, while Rac1 activation appears to be opposite from light off in nocturnal rats. From an endogenous melatonin viewpoint, we speculate that endogenous melatonin inhibits Rac1 activity in rats. How to deal with these inconsistent results from exogenous and endogenous melatonin is still unclear? Exogenous melatonin treatment during the day mimics the nighttime suppression of memory formation in the diurnal zebrafish (Rawashdeh et al., 2007). Consistent with our result, melatonin administration during the night impaired the better contextual fear memory in rats (Figure 4C). And Rac1 activation inhibitor NSC23766 counteracted the effect of melatonin, which indicated that increased hippocampal Rac1 activity underlies the impairment of contextual fear memory caused by melatonin. However, melatonin administration during the day does not worsen daytime memory, indicating by without affecting acquisition and retrieval (Figure 4B). We speculated that the ceiling effect limits the increased effect of melatonin on Rac1 activation; exogenous melatonin cannot further increase Rac1 activation when it is at a higher level during the daytime. Other studies reported that exogenous melatonin (2.5 mg/kg) impaired the acquisition but not the expression of contextual fear in rats during daytime (Yang et al., 2013). Melatonin facilitates extinction, but not acquisition or expression of conditional cued fear in rats (Huang et al., 2014). These discrepancies in results may have been created by different cognition tasks, training protocols, or melatonin dosage. In human umbilical vein endothelial cells, melatonin inhibited hypoxia-activated Rac1 activation mediated through ERK (Yang et al., 2014), while melatonin enhanced Rac1 activation in human germ cells (Noyes et al., 2021). We first reported the relationship between melatonin and Rac1 activity in the central nervous system. Here, we only administrate the systemic delivery of melatonin instead of injecting it locally into the hippocampus. However, many questions are worth further investigation in future studies. How exogenous melatonin regulates Rac1 activity both in nocturnal rats and diurnal tree shrews during their inactive and active period, respectively? Furthermore, the mechanism underlying the interaction between melatonin and Rac1 is still unclear. The underlying mechanisms are involved in changes in neuronal excitability, decreasing synaptic plasticity and decreasing neurogenesis in the hippocampus (Tononi and Cirelli, 2014; Kreutzmann et al., 2015; Brownlow et al., 2020).

We first demonstrated that hippocampal Rac1 activity altered depending on the time of day in both diurnal tree shrews and nocturnal rats. Time of day-dependent hippocampal Rac1 activation alteration modulates the efficiency of context memory in rats, which provides the first piece of evidence for understanding the daytime change of memory efficiency from the forgetting viewpoint. Interestingly, we find that exogenous melatonin administration can increase hippocampal Rac1 activation in rats, which may explicate the impairment effect of endogenous melatonin on contextual fear memory at nighttime by promoting forgetting. These results provide new insight into the function of forgetting in learning and memory.

## Data Availability Statement

The original contributions presented in this study are included in the article/Supplementary Material, further inquiries can be directed to the corresponding authors.

## Ethics Statement

The animal study was reviewed and approved by the Animal Care and Use Committee of Kunming Institute of Zoology, Chinese Academy of Sciences. Written informed consent was obtained from the owners for the participation of their animals in this study.

## Author Contributions

LJ, RM, and LX designed the study. LJ, CL, CM, BZ, YY, and QZ performed the experiments. LX and RM supervised the design and experiments. LJ and RM wrote the manuscript with the help of CL. All authors contributed to the article and approved the submitted version.

## Conflict of Interest

The authors declare that the research was conducted in the absence of any commercial or financial relationships that could be construed as a potential conflict of interest.

## Publisher’s Note

All claims expressed in this article are solely those of the authors and do not necessarily represent those of their affiliated organizations, or those of the publisher, the editors and the reviewers. Any product that may be evaluated in this article, or claim that may be made by its manufacturer, is not guaranteed or endorsed by the publisher.
