# Supplementary material for: Time of Day-Dependent Alteration of Hippocampal Rac1 Activation Regulates Contextual Fear Memory in Rats
Source: Front Mol Neurosci. 2022 Jun 16;15:871679. doi: 10.3389/fnmol.2022.871679 (PMC9245039; doi:10.3389/fnmol.2022.871679)
Supplement: Supplementary file 1 [file Data_Sheet_1.pdf]

Supplementary Material

1. Supplementary Figures

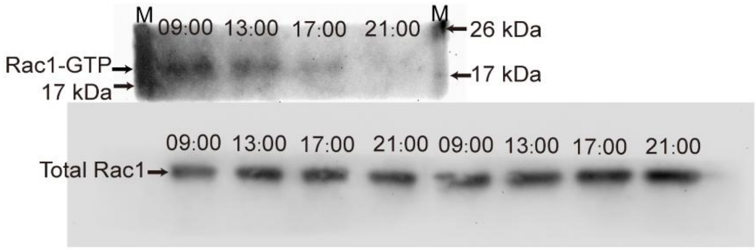

Supplementary Figure 1. Full scan of the entire original gels of Fig. 1B. M is abbreviation for maker.

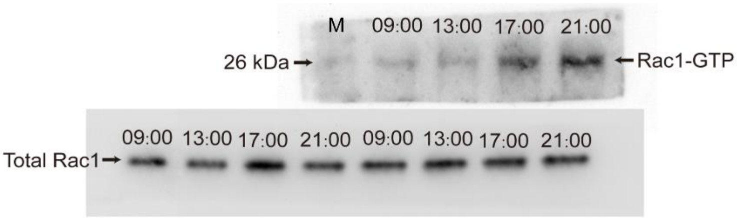

Supplementary Figure 2. Full scan of the entire original gels of Fig. 1C.

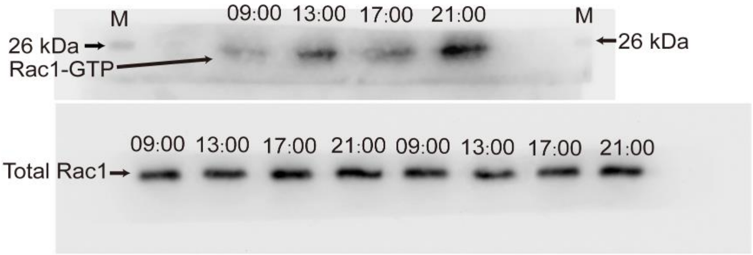

Supplementary Figure 3. Full scan of the entire original gels of Fig. 3B.

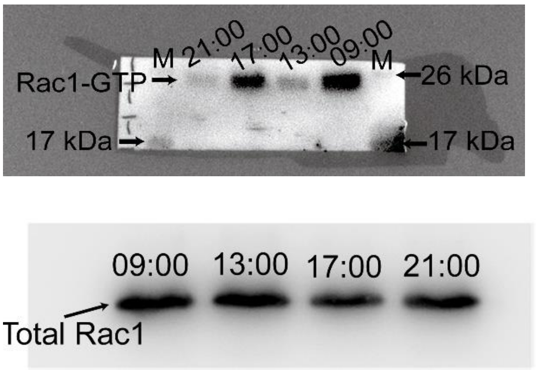

Supplementary Figure 4. Full scan of the entire original gels of Fig. 3C.

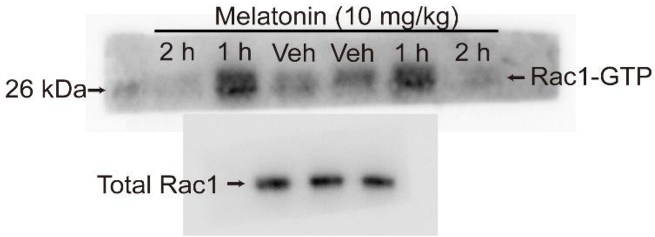

Supplementary Figure 5. Full scan of the entire original gels of Fig. 4A.
